# Case Series of Neonatal Extravasation Injury: Importance of Early Identification and Management

**DOI:** 10.7759/cureus.21179

**Published:** 2022-01-12

**Authors:** Chun Kai Yew, Siti Fatimah Noor Mat Johar, Wee Yi Lim

**Affiliations:** 1 Reconstructive Science Unit, Hospital Universiti Sains Malaysia, Kota Bharu, MYS; 2 Plastic and Reconstructive Surgery, Hospital Queen Elizabeth, Kota Kinabalu, MYS

**Keywords:** necrosis, newborn, extravasation of diagnostic and therapeutic materials, dressing, cannula, gault technique, saline flush-out, intravenous fluid, neonate, extravasation injury

## Abstract

Extravasation injury is a common iatrogenic injury, especially in neonates. Intravenous access is essential in neonatal care, but neonatal extravasation injury is associated with severe morbidity. We present three cases of neonatal extravasation injuries with varying presentations, etiological agents, and timing of management. It shows that extravasation injuries treated with the saline flush-out technique and timely intervention have a superior outcome with almost immediate resolution and subsequent healing with no scars. This is in stark contrast with the lesions treated conservatively with dressings that took more time to heal. We are reminded to be vigilant with infusion therapies and the importance of early detection and prompt treatment in neonatal extravasation injuries.

## Introduction

Extravasation injury is a common iatrogenic injury in the neonatal intensive care unit. It is associated with severe morbidity in neonates. The use of intravenous (IV) access to provide medication and nutrition is essential, and the fragile and minuscule neonatal veins make it challenging to keep the IV lines in situ. Furthermore, some IV accesses are required for long periods. In addition, with the neonate's inability to communicate, the risk of extravasation injury is apparent. The prevalence of extravasation injury among neonates was 38 per 1,000 neonates, while the incidence among neonates was 12.6 per 100 peripheral IV catheter days [1]. Another study of 1,409 neonates reported a severe injury rate of 2.4%, with total parenteral nutrition (TPN) solution being involved in most cases [2]. Different methods of treatment are available, and they can be divided into surgical and non-surgical therapies. Non-surgical management includes pain relief, cold compressions, and dressing. On the other hand, surgical management options include hyaluronidase injections, saline flush-out technique, liposuction, and surgical excision.

We present three cases of neonatal extravasation injuries with varying presentations, aetiological agents, and timing of management.

## Case presentation

Case 1

A two-day-old baby girl, born preterm at 36 weeks of gestation, was given human albumin infusion IVly via branula over the left antecubital fossa while receiving treatment in the neonatal intensive care unit. She started developing redness and swelling over the branula site. The redness progressed into blisters that became necrotic and ulcerated three days later. A referral to the Plastic Reconstructive Surgery team was only made on post-trauma day 8.

**Figure 1:**
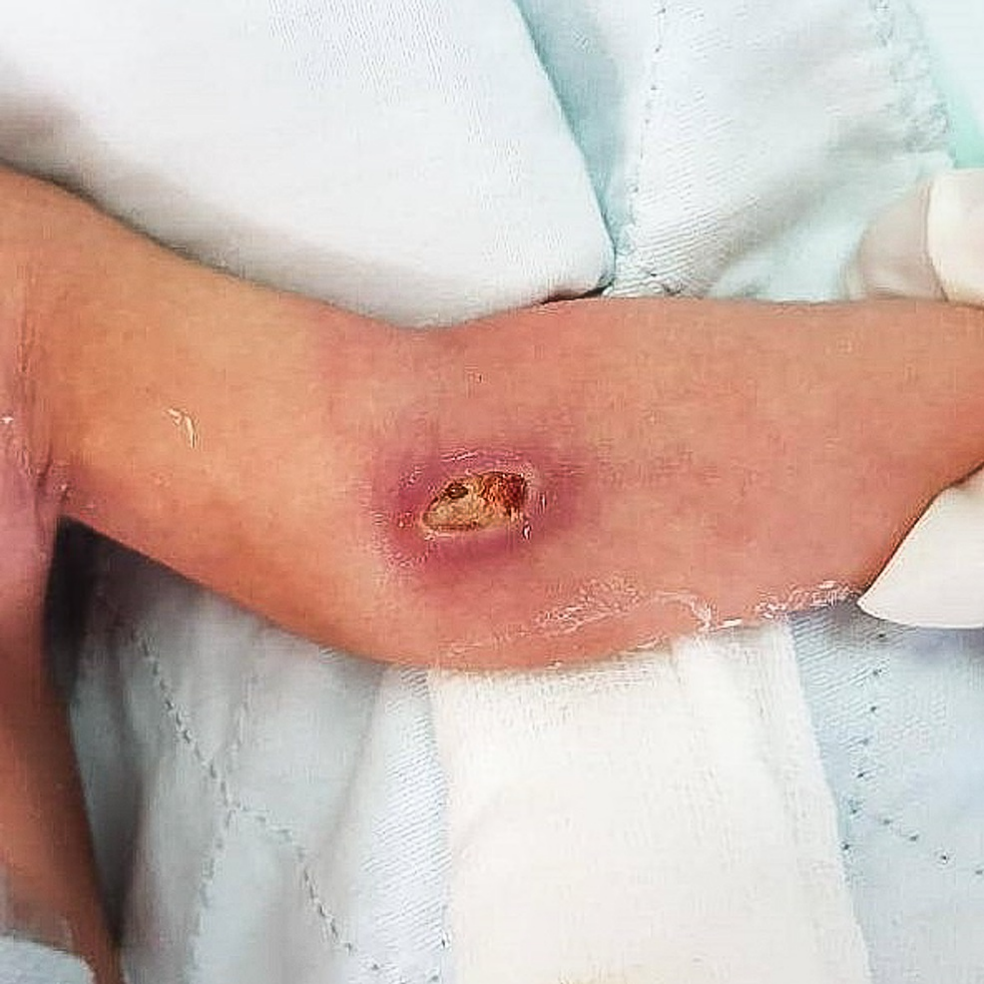
Antecubital fossa full-thickness skin loss with surrounding erythematous wound edge.

Upon examination, there was a left antecubital fossa wound, with 1cm x 0.5cm full-thickness skin loss with surrounding erythematous wound edge (Figure 1). A superficial thrombosed vessel could be felt, which was likely the previous cannulation vessel. The brachial artery could be felt pulsating nearby, and distal circulation was intact.

The child was treated as a case of extravasation injury over the left antecubital fossa with causative agent human albumin infusion. She was started on daily gel and transparent film dressing.

**Figure 2:**
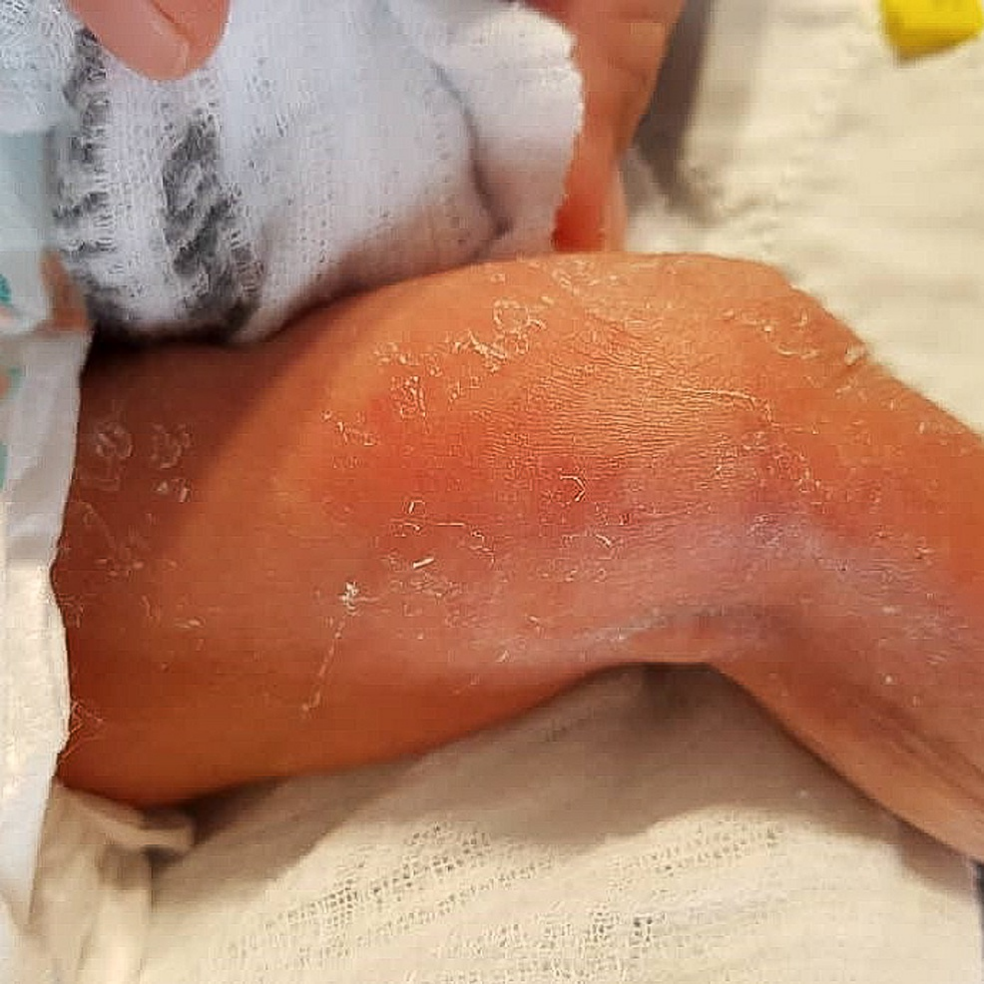
Area of induration over the left distal medial thigh, with redness and warmth.

Unfortunately, on post-trauma day 9, we noticed redness and swelling over her left distal medial thigh where an IV infusion of human albumin was given, and the branula was already removed. She developed a 3cm x 1.5cm area of induration with redness and warmth (Figure 2).

We proceeded with the saline flush-out technique. Multiple puncture wounds were made over the induration, and irrigation with normal saline was done. Over two days, the inflamed induration over the left distal medial thigh resolved with residual bruising. At the same time, the left antecubital fossa wound was healing slowly. The child was discharged home after her acute issues were resolved, with her dressing continued in the outpatient department.

**Figure 3:**
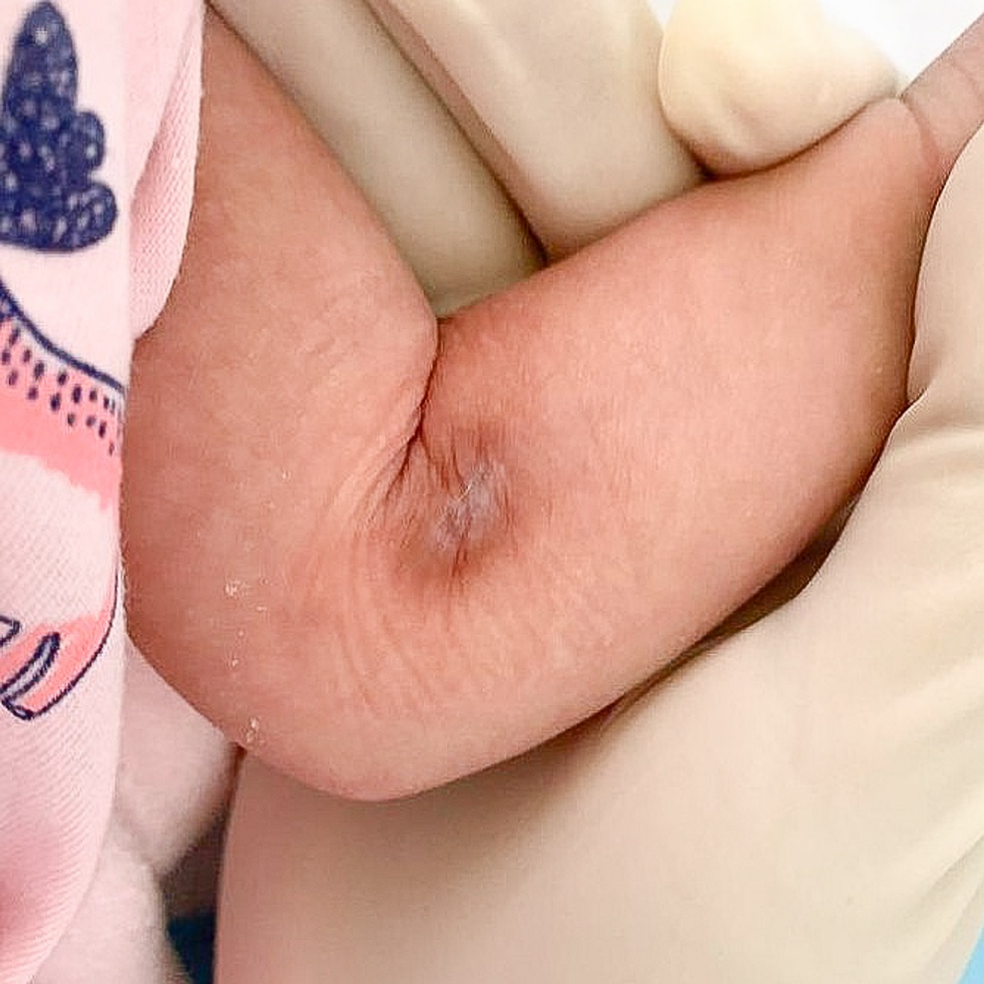
Antecubital fossa wound healed via secondary intention after six weeks.

**Figure 4:**
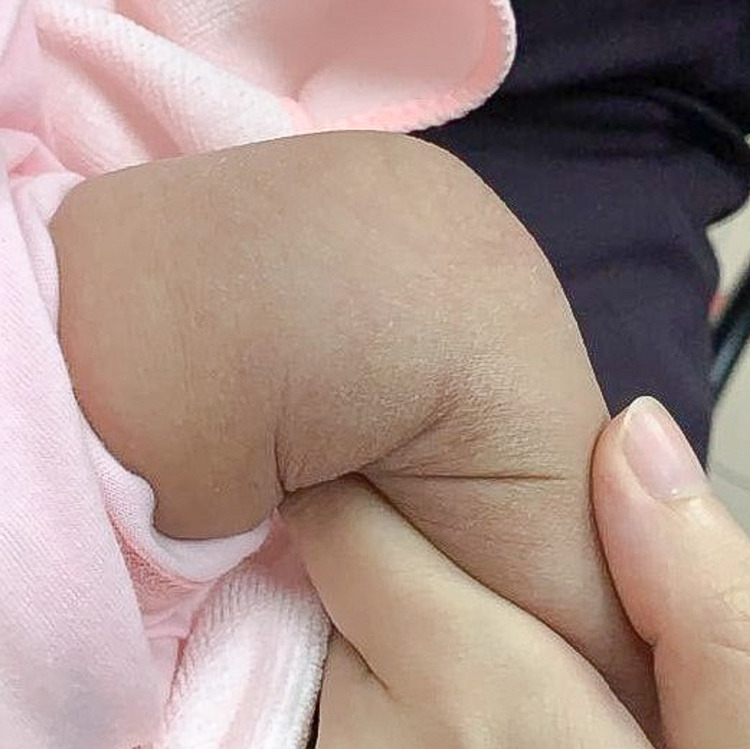
Left distal medial thigh puncture wound healed with no visible scars.

She came back for clinic review at six weeks of age. Her antecubital fossa wound was healed by secondary intention (Figure 3), whereas her left distal thigh puncture wounds were healed with no visible scars (Figure 4).

Case 2

**Figure 5:**
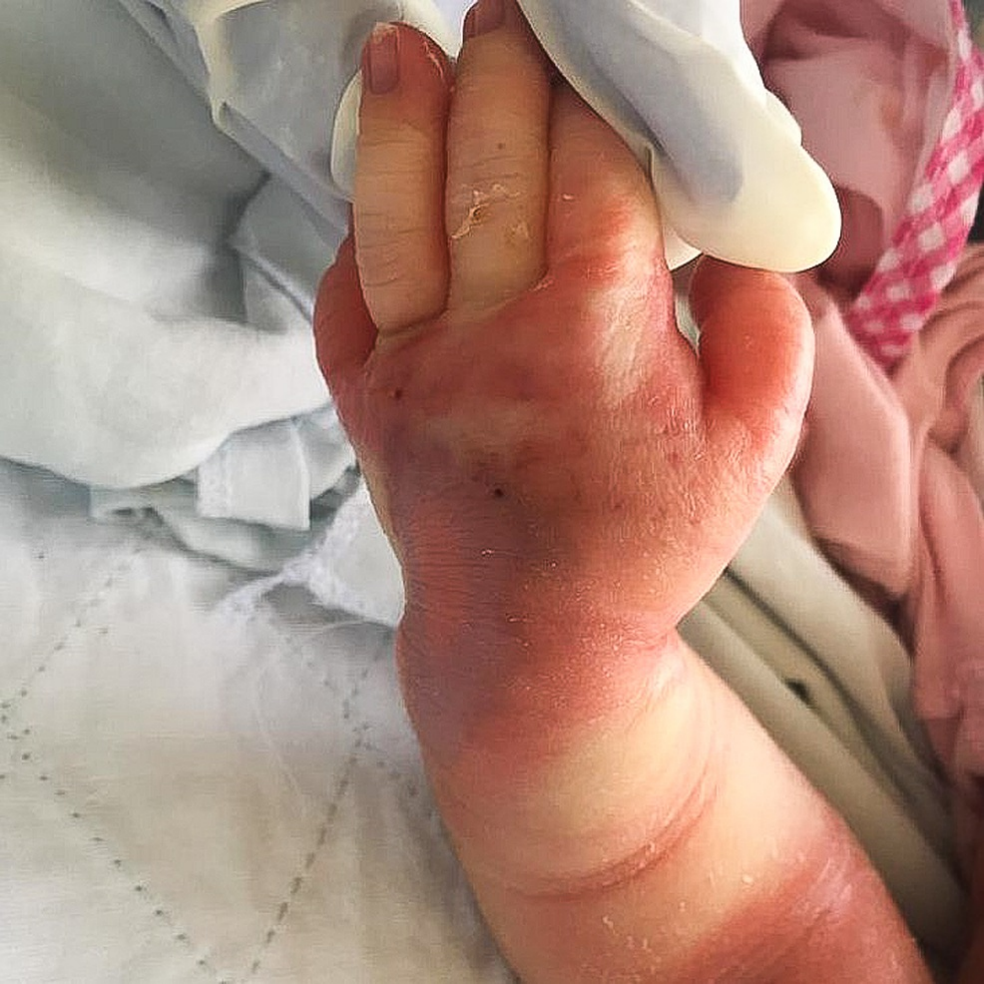
Left hand swollen with an area of discoloration at dorsum and surrounding erythema.

**Figure 6:**
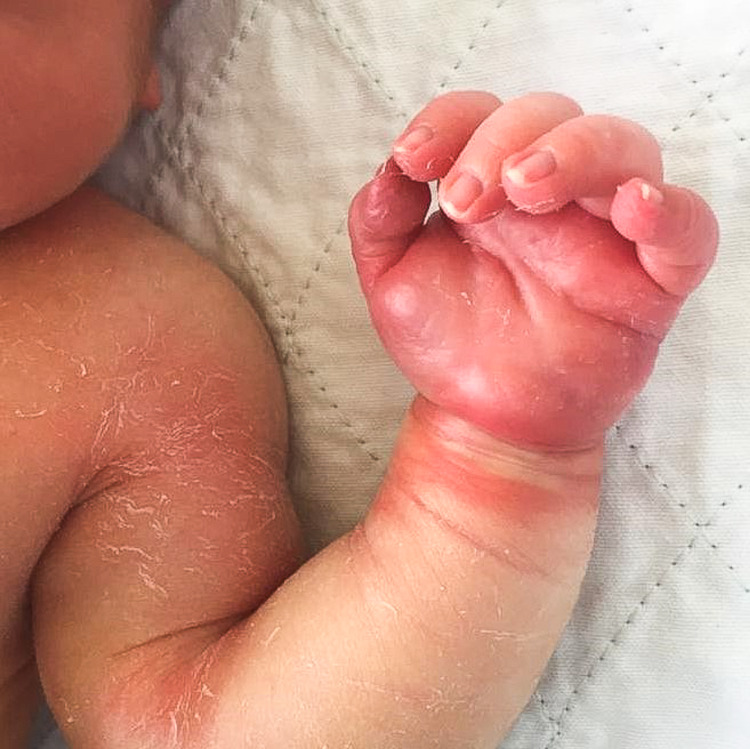
Left palm grossly swollen with erythema.

A one-day-old baby girl, born term at 38 weeks of gestation, received an IV infusion of dextrose 10% via left-hand dorsum branula while being treated in the COVID cohort section of the neonatal ward. The maintenance drip was running overnight for eight hours, and the primary team noted reddish discoloration over the branula site with the left hand grossly swollen the following morning (Figures 5, 6). The branula was removed, and the wound progressed into a necrotic patch. The patient was referred to the Plastic Reconstructive Surgery team on post-trauma day 3.

**Figure 7:**
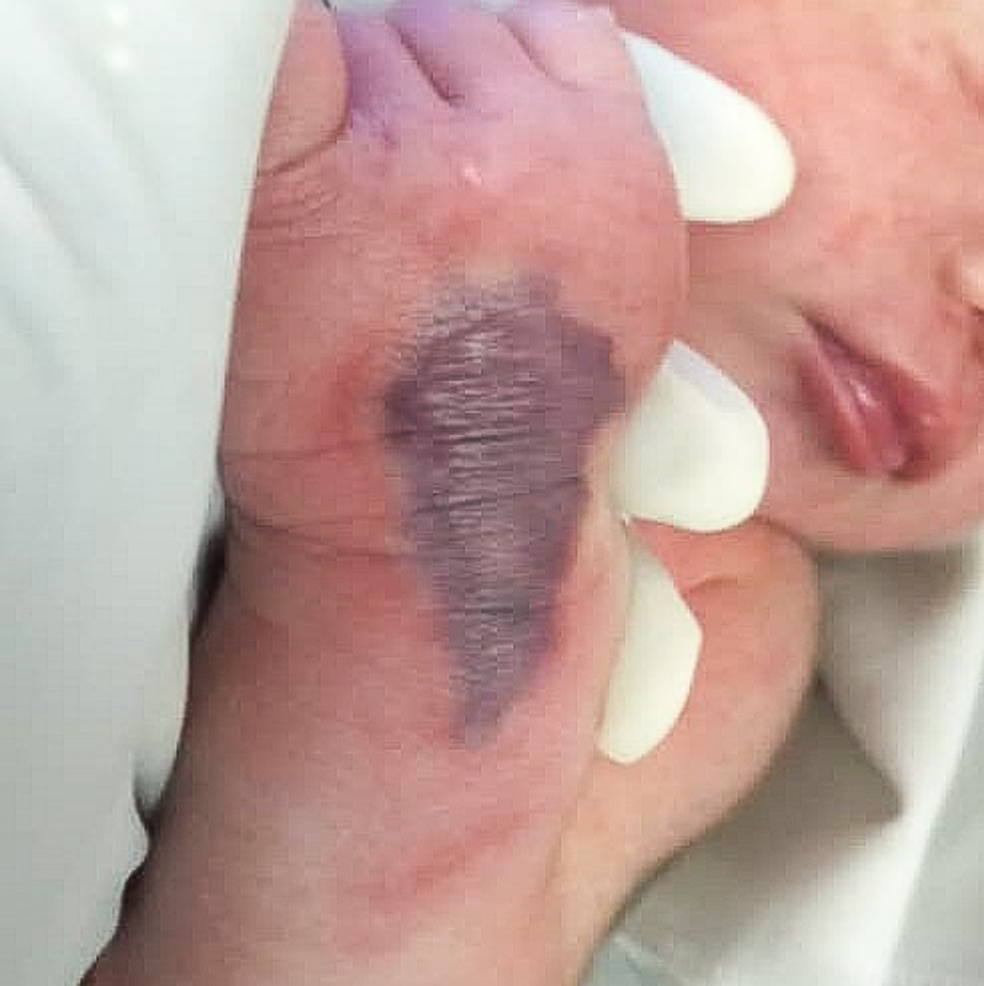
Area of necrosis demarcated with minimal intact blisters and surrounding erythema.

Upon examination, the left-hand dorsum revealed a 3.5cm x 1.5cm necrotic patch with minimal intact blisters (Figure 7). The circulation over all five fingers was intact. The child was started on daily normal saline and Bactigras® dressing (Smith & Nephew, Hull, UK).

**Figure 8:**
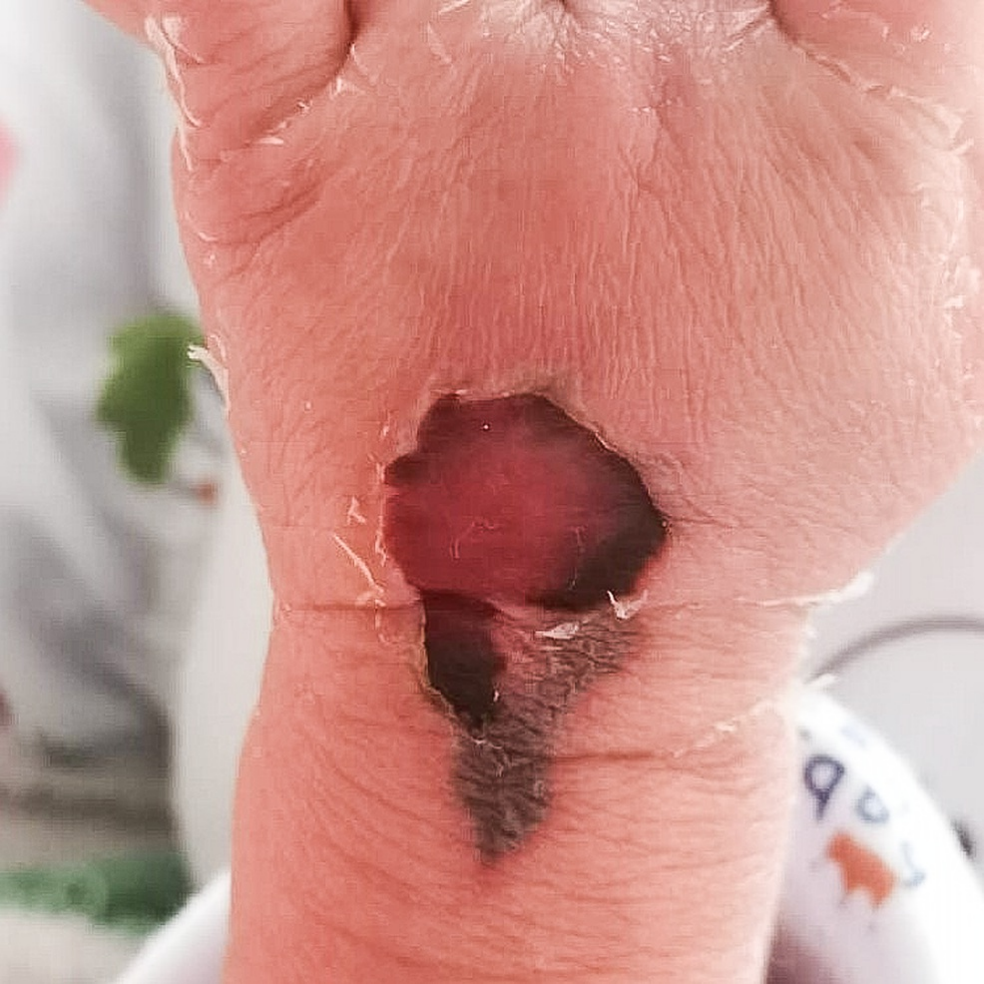
Dry necrotic patch over the left-hand dorsum.

By post-trauma day 6, the necrotic area over the dorsum of the left hand became dry and reduced in size to 1.5cm x 1cm (Figure 8). The dressing was continued, and the wound healed uneventfully.

Case 3

A four-day-old baby boy, born term at 37 weeks of gestation, was admitted to the neonatal intensive care unit and started on TPN via a branula over the left-hand dorsum. Two hours after the infusion, left-hand swelling was noted. The TPN infusion was stopped immediately. Aspiration was attempted over the branula, but no content was aspirated. The branula was removed, and the case was referred to the Plastic Reconstructive Surgery team.

**Figure 9:**
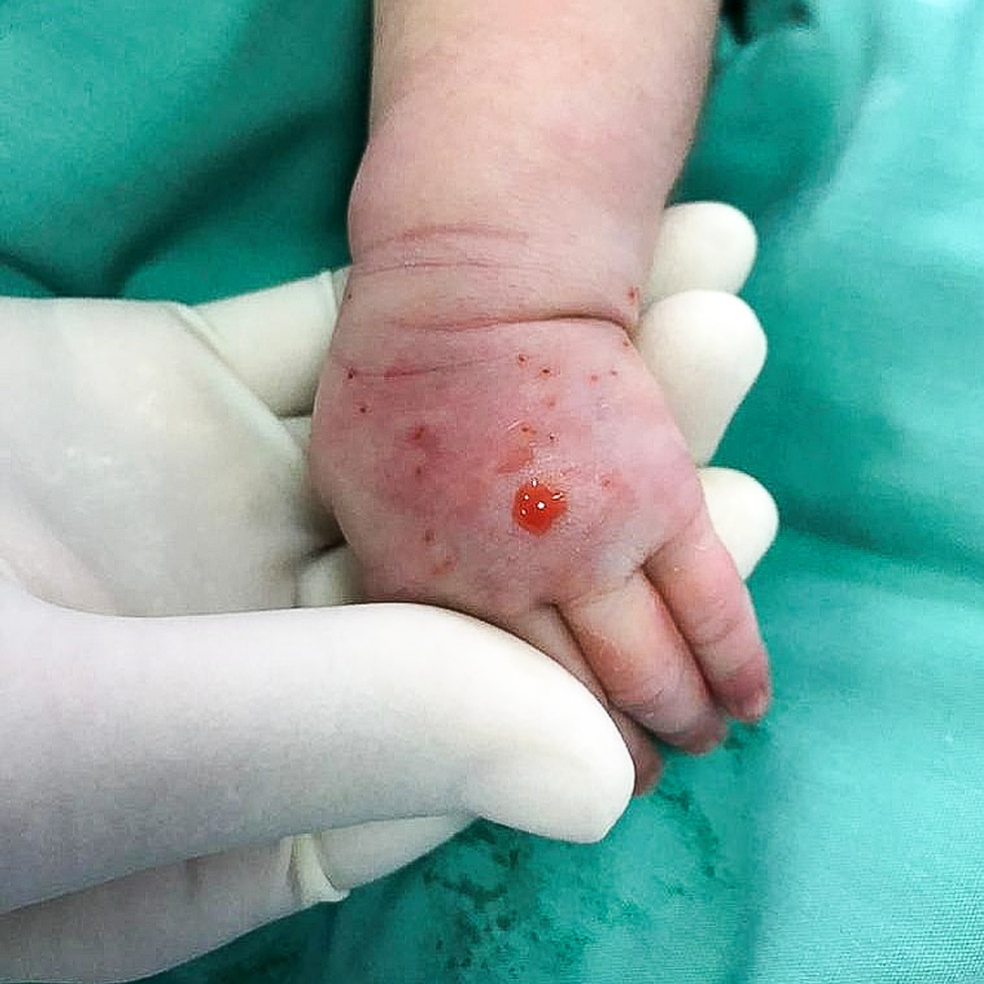
Left-hand dorsum swollen, erythematous with hemoserous discharge.

Upon examination, the left-hand dorsum was swollen and erythematous up to the wrist, with yellowish hemoserous fluid oozing from the branula site (Figure 9). Multiple puncture sites were noted on the left-hand dorsum, indicating multiple attempts at venepuncture and line setting. All fingers were pink, and the distal circulation was intact.

**Figure 10:**
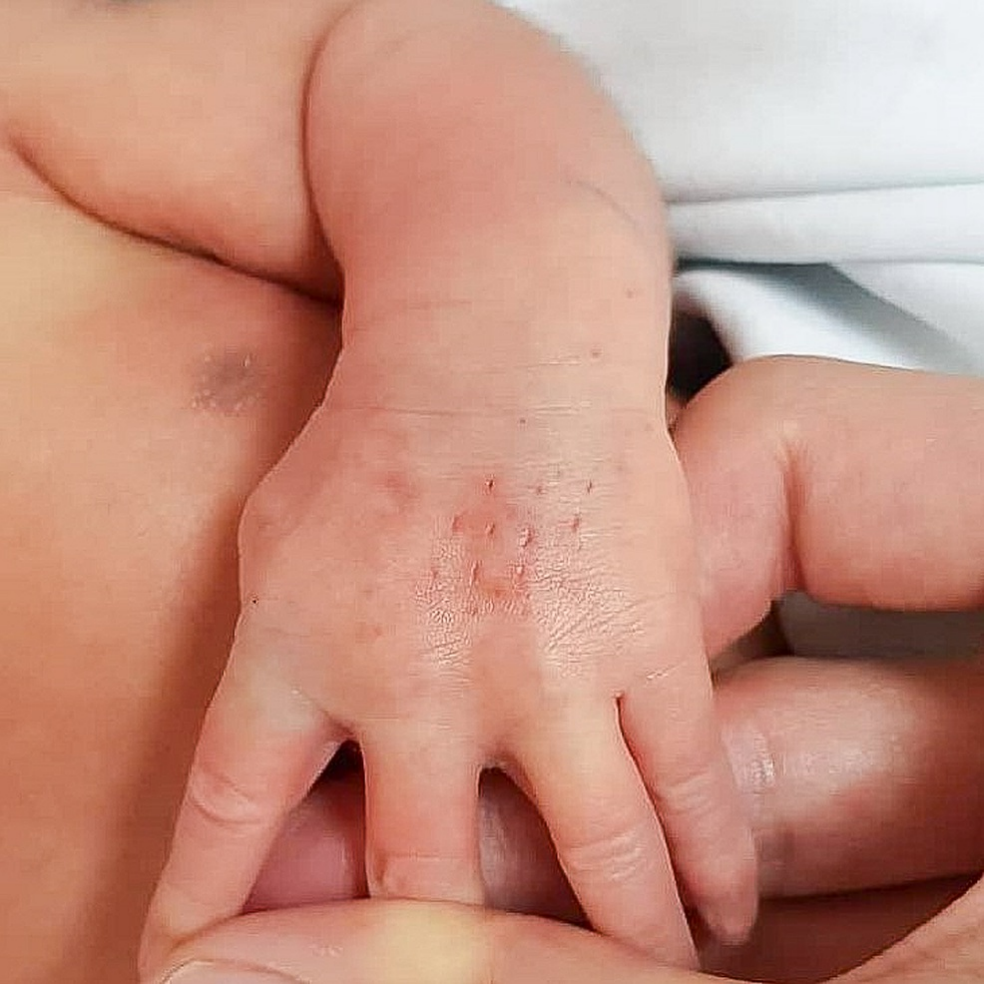
Swelling resolved with minimal residual erythema.

The saline flush-out technique was done for this case as well. Multiple puncture wounds were made, and irrigation was done with normal saline. The dressing was done with Bactigras and a bandage. Over two days, the left-hand dorsum swelling resolved with residual erythema (Figure 10). There was no discharge from the puncture sites. The daily dressing was continued until the erythema resolved and the puncture wounds healed.

## Discussion

Extravasation injury is defined as accidental solution infiltration from a dislodged branula into the subcutaneous tissue. The tissue damage occurs due to solution osmolality, tissue toxicity, vasoconstrictor properties, infusion pressure, and anatomical peculiarities. Depending on the nature and volume of the infiltrating solution, the extravasation may go unnoticed or cause severe damage leading to skin loss, infection, nerve or tendon involvement, and even compartment syndrome requiring amputation [3]. The common site of extravasation coincides with the common areas of branula insertion, namely over the dorsum of the hand and foot, antecubital fossa, and ankle. Expectedly, more extravasations occur at night and often go unnoticed [4], as seen in case 2.

Several factors contribute to the incidence of neonatal extravasation. Neonates present with smaller caliber vessels and poor venous integrity, especially preterm and low birth weight babies. There is a greater chance of vasoconstriction and capillary leakage. The lack of tissue support compared to their older counterparts makes line insertion more difficult. Lastly, there is the inability to report pain. All this adds up to a greater chance of injury in this group of patients [5]. The mechanism of injection plays a crucial role as well. The use of infusion pumps leads to a higher risk of trauma to the vessels. Branulas near joint areas and lower limbs are more susceptible to dislodge due to movement. Multiple previous venepunctures may compromise the venous wall integrity [5], which might have been one of the reasons for extravasation in case 3.

The extravasant, being the etiological agent, also determines the extent of tissue injury based on its volume injected, the concentration of the solution, and physiochemical properties of the injected agent. The risk of extravasation increases depending on the osmolarity, pH levels, and pharmacological effects of the solution. Thus, several recommendations have been made regarding the safety of peripheral infusions [6]. The American Academy of Pediatrics Committee on Nutrition recommends that only solutions with osmolarity less than 900 mOsm be administered peripherally, whereas the Infusion Nurses Society recommends limiting peripherally administered solutions to less than 500 mOsm. Solutions outside the pH range of 5 to 9 also have an increased risk of inflammation and vascular injury. Vasoactive medications produce vasoconstriction, which increases the risk of backflow and reduces local blood flow via constriction of capillary beds, resulting in local tissue ischemic injury.

The extravasant can be classified into vesicants, irritants, and neutral agents, depending on its cytotoxicity levels [7]. Vesicants are solutions that result in tissue necrosis when infused into the tissue, with common examples consisting of DNA binding chemotherapy drugs such as doxorubicin. TPN used in case 3 is considered a vesicant as it is a hyperosmolar solution and acidic in nature, with a high concentration of ionic substances [8]. Dextrose 10% solution used in case 2 is also a vesicant as it is hyperosmolar and acidic [9]. Irritants are medications that cause inflammation, pain, or irritation at the extravasation site. They are typically self-limiting without long-term sequelae. Human albumin solution is considered an irritant [7], but the extent of tissue damage varies depending on other factors mentioned above, such as volume and pressure injected. This can explain the different presentations in case 1, with a full-thickness skin loss in the antecubital fossa and only inflamed induration at the left medial thigh, despite both extravasants being the same culprit.

Presentation of extravasation injury includes pain, swelling, erythema, tenderness, local blistering, mottling, induration, non-blanching skin, and ulceration, which is a late sign. Blistering and induration, that persist for more than 24 hours, signify a severe extravasation injury and the risk of developing ulceration. These signs are commonly seen in thrombophlebitis and skin infections, and the diagnosis will be missed if the clinician is not aware. Typical progression of an extravasation injury of a vesicant starts with primary vasodilatation and sludging of red cells as early as two to four hours after injury, with vascular endothelial degenerative changes happening over the first to second day, and later necrotic lesions appear [5]. These changes can be well appreciated from Figures 5-8.

The severity of extravasation injuries was classified by Loth and Eversmann into mild, moderate, and severe [7]. Mild and moderate injuries involve a small volume of extravasant, leading to local inflammatory reactions without blistering. On the other hand, severe injuries are caused by a large volume of extravasant, typically vesicant agents, resulting in marked swelling, inflammation, blistering, and ulceration. The classification aids in management as mild-to-moderate injury usually only requires symptom relief and non-surgical management, whereas severe injury warrants active intervention. Necrosis interval was introduced wherein timely surgical intervention may prevent further tissue necrosis. The necrosis interval for vasopressors is four to six hours, radiological contrasts at six hours, and chemotherapy medications up to 72 hours.

Non-surgical management of extravasation injury includes pain relief, cold compressions, and dressing. Cold compression here serves to deactivate and decrease the spread of the extravasant and slow down the cellular metabolic rate. Only 11% to 21% of all extravasation injuries required surgical intervention [7], which is the primary rationale behind the conservative approach. However, cold compression should be used cautiously when handling the extravasation of chemotherapy agents. Cold compression is suggested for use in DNA-binding drugs such as anthracyclines and alkylating agents, where cold will result in vasoconstriction, minimizing the spread of the drug in the tissues and allowing time for the local vascular and lymphatic systems to disperse the drug. Heat compression is suggested for non-DNA binding drugs such as vinca alkaloids and taxanes. The heat will cause vasodilation, increasing drug distribution and absorption, aiding its dispersal. Hand elevation is also vital to facilitate venous and lymphatic return in order to reduce edema. Antidotes are available to treat extravasation injury of specific agents, but they are used only in cases involving vesicants or large volume extravasations.

Surgical management is employed for severe extravasation injuries, including hyaluronidase injections, saline flush-out technique, liposuction, and definitive surgical excision. When used in the first hour post-trauma, subcutaneous hyaluronidase injections significantly reduced the area of necrosis. The interstitial fluid barrier composed of hyaluronic acid is broken down by hyaluronidase, which facilitates the dispersion of the extravasant into the surrounding tissues, decreasing the concentration effects of the agent while exposing it to more capillary beds to increase reabsorption and removal of the extravasant. Current recommendations suggest 1-mL hyaluronidase injection for each milliliter of extravasant in a clockwise manner of the targeted area [5]. Gault's saline flush-out technique was described in 1993 [3]. The original technique injects 1,500-unit hyaluronidase into the area of injury, followed by four stab incisions at the area's periphery, and then saline is irrigated through the subcutaneous tissue. A total of 500mL of fluid is recommended, and the technique is ideally performed within six hours of the incident, with reduced efficacy with time but may still have benefit up to 24 hours. It was reported that 86 patients who received treatment within 24 hours healed with no soft tissue loss. Out of 18 cytotoxic extravasation injuries treated with hyaluronidase and saline flush-out technique, 17 healed spontaneously, and it was also reported to be successful in infants with parenteral nutrition extravasation. This case series shows that extravasation injuries treated with the saline flush-out technique and timely intervention have a superior outcome with almost immediate resolution and subsequent healing with no scars. This is in stark contrast with the lesions treated conservatively with dressings that took much longer to heal.

Algorithms have been developed in various neonatal units to manage extravasation injury [6], but all rely on one crucial step: identifying signs and symptoms. Early diagnosis and prompt treatment yield better results for reasons explained above, as seen in various reports and our current case series. However, there is often a natural tendency to delay urgent surgical consultation [3], and referral is only made after the necrosis interval. Failure to detect and treat extravasation injury leads to tissue necrosis, resulting in more extended hospital stay, secondary infection, and higher morbidities.

## Conclusions

Our report highlights the importance of early detection and prompt treatment of neonatal extravasation injuries. We have to be vigilant with infusion therapies and the solutions given, beginning from the process of cannulation to regular monitoring during infusion and strict protocols on the identification and management of extravasation injuries. If treatment is delayed, the child will have to endure a long journey to recovery with increased pain and morbidity. In the event of extensive necrosis and tissue loss, the worst-case scenario might even end with amputation.
